# A Freestanding Single‐Wall Carbon Nanotube Film Decorated with N‐Doped Carbon‐Encapsulated Ni Nanoparticles as a Bifunctional Electrocatalyst for Overall Water Splitting

**DOI:** 10.1002/advs.201802177

**Published:** 2019-04-19

**Authors:** Abdul Majeed, Peng‐Xiang Hou, Feng Zhang, Hassina Tabassum, Xin Li, Guo‐Xian Li, Chang Liu, Hui‐Ming Cheng

**Affiliations:** ^1^ Shenyang National Laboratory for Materials Science Institute of Metal Research (IMR) Chinese Academy of Sciences 72 Wenhua Road Shenyang 110016 China; ^2^ University of Chinese Academy of Sciences (UCAS) 19 A Yuquan Road Beijing 100049 China; ^3^ School of Materials Science and Engineering University of Science and Technology of China Hefei 110016 P. R. China; ^4^ Beijing Key Laboratory for Theory and Technology of Advanced Battery Materials Department of Materials Science and Engineering College of Engineering Peking University Beijing 100871 China; ^5^ Tsinghua‐Berkeley Shenzhen Institute (TBSI) Tsinghua University Shenzhen 518055 China

**Keywords:** bifunctional electrocatalysts, carbon‐encapsulated nanoparticles, freestanding films, Ni nanoparticles, overall water splitting, single‐walled carbon nanotubes

## Abstract

Noble‐metal free, cost‐effective, and highly stable catalysts with efficient activity for both the hydrogen evolution reaction (HER) and the oxygen evolution reaction (OER) have attracted tremendous research interest in recent years. Here, a flexible, self‐standing hybrid film comprising a N‐doped single‐wall carbon nanotube (SWCNT) network on which are anchored Ni nanoparticles encapsulated by a monolayer of N‐doped carbon (NCNi) is reported. The films are prepared by floating catalyst chemical vapor deposition followed by NH_3_ treatment. The material obtained at optimum conditions shows excellent bifunctional electrocatalytic activity in alkaline media with low overpotentials of 190 and 270 mV for HER and OER, respectively, to reach a current density of 10 mA cm^−2^. A current density of 10 mA cm^−2^ at 1.57 V is achieved when this freestanding and binder‐free rod‐shaped NCNi/SWCNT assembly is used as cathode and anode in 1 m KOH solution for overall water splitting, presenting one of the best values reported to date.

## 1 Introduction

The rapidly increasing demand for energy due to global industrialization and environmental pollution caused by fossil fuel consumption has made it essential to find new and clean energy sources. Hydrogen is a clean, renewable, and sustainable energy source and the electrochemical splitting of water is considered one of the most reliable and efficient ways for the production of hydrogen.^1^, ^2^, ^3^ Electrochemical water splitting is based on two half reactions: the hydrogen evolution reaction (HER) and the oxygen evolution reaction (OER).^4^, ^5^ Owing to the intrinsic sluggish kinetics of these reactions, electrocatalysts are necessary to stimulate and accelerate these processes. Currently, the state‐of‐the‐art catalysts are based on noble metals, including Pt, Ir, and Ru. However, their low abundance, high cost, and nonsustainability have seriously impeded their use in water splitting and fuel cells, air–metal batteries and other related energy storage/conversion devices. Therefore, tremendous effort has been devoted to developing low‐cost, high‐efficiency, stable, and earth‐abundant electrocatalysts.^6^ To date, several nonprecious electrocatalysts based on nanostructured transition metals (Ni, Fe, and Co) and their derivatives (oxides, phosphides, and hydroxides), and nitrogen‐functionalized carbon/transition metal composites have been reported to be effective catalysts for the HER and OER.^7^, ^8^, ^9^, ^10^ Among them, Ni‐based nanostructures, including Ni alloys and Ni phosphides have attracted intense research interest due to their high catalytic activity and stability. Carbon nanotubes (CNTs) are considered an ideal heteroatom support for catalysis owing to their high conductivity, large surface area, and good chemical stability.^11^, ^12^, ^13^, ^14^ Therefore, various hybrid structures consisting of CNTs and nanometals have been prepared for electrocatalysis. However, most of these are active for only HER or OER and show catalytic activity in different electrolysis media.^15^, ^16^, ^17^, ^18^ Therefore, it is difficult to use such catalysts in one electrolyte for overall water splitting due to the mismatch of electrolyte pH. Consequently, it is highly desirable to synthesize electrocatalysts that function simultaneously for both HER and OER in same electrolyte. In addition, it is also a challenge to prevent the oxidation and aggregation of these metal nanostructures, which may seriously degrade their overall catalytic activity. To solve these problems and to augment the electrocatalytic activity, many strategies have been developed including reducing the particle size to improve reactivity, enclosing the metal nanoparticles with a carbon layer and coupling the metallic species with heteroatom‐doped carbon materials.^19^, ^20^, ^21^, ^22^ Furthermore, nonconductive binder adhesives which are commonly used to attach the catalyst materials to conductive supports increase the interfacial resistance and hence decrease the overall conductivity.^23^, ^24^, ^25^ Therefore, obtaining a support‐free single‐walled carbon nanotube (SWCNT) film loaded with abundant, stable, and small metal nanoparticles is highly needed to achieve bifunctional electrocatalytic activity.

In our previous work, we synthesized an SWCNT film on which were anchored Ni nanoparticles encapsulated by a monolayer of carbon (CNi/SWCNT) by a floating catalyst chemical vapor deposition (FCCVD) method and demonstrated its supercapacitive property after oxidizing Ni to NiO. Here, we further report the preparation of a nitrogen‐doped CNi/SWCNT (NCNi/SWCNT) film for bifunctional HER and OER electrocatalysis. The N‐doping introduces additional active sites for the catalytic reaction by forming defective edges in the hybrid structure. At the same time the electronegativity of the nanocarbons was tailored by introducing heteroatoms. As a result the NCNi/SWCNT films were found to be highly efficient and stable for both HER and OER and hence for overall water splitting in alkaline media. Moreover, the SWCNT network in our NCNi/SWCNT freestanding films provides both electron transfer paths and acts as a support for loading Ni nanoparticles, avoiding the use of any nonconductive adhesive.

## 2 Results and Discussion

**Figure 1:**
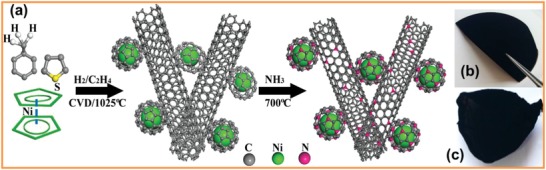
a) Schematic showing the preparation process of NCNi/SWCNT films. b,c) Typical optical images of the as‐prepared CNi/SWCNT film and NCNi/SWCNT film, respectively.

The preparation process of the NCNi/SWCNT film is schematically shown in **Figure** **1**a. Figure 1b,c shows typical optical images of as‐prepared CNi/SWCNT and NCNi/SWCNT films, respectively. There is no obvious difference observed for the films before and after NH_3_ treatment.

**Figure 2:**
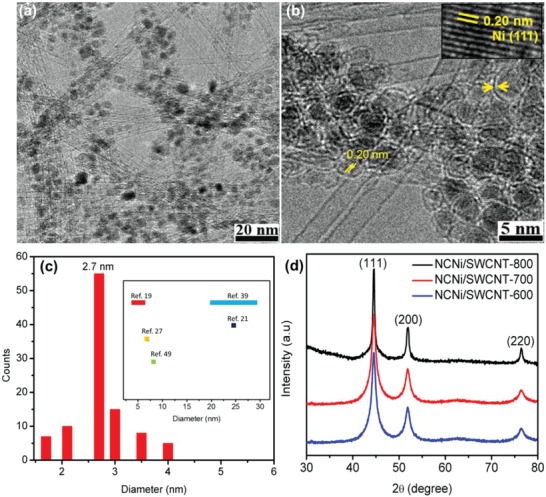
a,b) TEM images of the NCNi/SWCNT‐700 film. The inset in (b) shows lattice fringes of an encapsulated Ni nanoparticle. c) Diameter distribution of Ni nanoparticles in the NCNi/SWCNT‐700 sample. The inset shows the mean diameters of recently reported carbon layer‐encapsulated metal nanoparticle electorcatalysts. d) XRD patterns of NCNi/SWCNT‐600, NCNi/SWCNT‐700, and NCNi/SWCNT‐800.

To identify microstructural changes after N doping, we performed transmission electron microscopy (TEM), Raman spectroscopy, X‐ray diffraction (XRD), and X‐ray photoelectron spectroscopy (XPS). TEM images of an NCNi/SWCNT‐700 film are shown in **Figure** **2**a,b, and TEM images of NCNi/SWCNT‐600 and NCNi/SWCNT‐800 films are shown in Figure S1 of the Supporting Information. No obvious difference was observed between these three samples, except that some of the carbon covering on the Ni particles had been destroyed/oxidized for the NCNi/SWCNT‐800 sample as indicated by arrows in Figure S1b of the Supporting Information. An entangled SWCNT network with small nanoparticles uniformly distributed and anchored on the surface of the SWCNTs was observed, which is similar to the morphology of the CNi/SWCNT films.^26^ High‐resolution TEM images show that the particles are covered with monolayer carbon. The inset in Figure 2b shows a magnified image of a metal particle with a fringe spacing of 0.20 nm, which is identified as the (111) plane of cubic metallic Ni.^27^ The particles diameters were measured from TEM images, and a histogram of the distribution is shown in Figure 2c. We can see that the size of most nanoparticles is in the range of 2–4 nm, and the mean diameter is 2.7 nm, which is similar to that of CNi/SWCNTs and much smaller than those of recently reported metal nanoparticles encapsulated by carbon for electrocatalysis (inset of Figure 2c). The small and uniform particle size would give the catalyst a high activity by providing a large exposed surface area and abundant active sites for catalyzing both OER and HER. The monolayer carbon‐cover on small Ni particles not only guarantees access of reactants to the Ni core but also prevents Ni particles from air‐oxidation and aggregation, which preserves their high electrocatalytic activity and long‐term stability.^28^, ^29^

**Figure 3:**
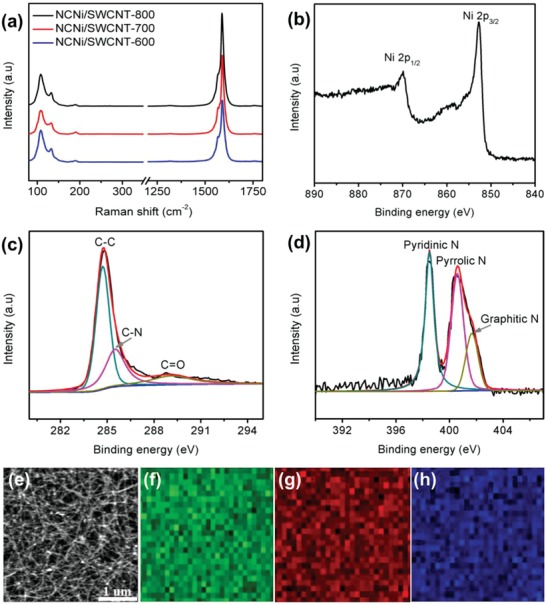
a) Raman spectra of three NCNi/SWCNT films obtained at different temperatures. b–d) High‐resolution XPS Ni 2p, C 1s, and N 1s spectra of the NCNi/SWCNT‐700 sample. e–h) SEM image of NCNi/SWCNT‐700 and corresponding EDS elemental maps for f) C, g) N, and h) Ni.

To further reveal the crystalline structure of the NCNi/SWCNT samples, X‐ray diffraction analysis was performed. As shown in Figure 2d, three sharp diffraction peaks were observed in all the samples and they could be assigned to the (111), (200), and (220) planes of cubic Ni (JCPDS No. 04–0850), which is consistent with TEM observations. Therefore, both TEM and XRD indicate that the Ni particles in NCNi/SWCNT films remained crystalline even after the NH_3_ treatment. Laser Raman spectra of the NCNi/SWCNT films are shown in **Figure** **3**a. Radial breathing mode peaks in the range of 80–150 cm^−1^ were detected for all the samples heat treated at different temeperatures.^30^, ^31^ However, the G/D intensity ratios of NCNi/SWCNT‐600, ‐700, and ‐800 films decreased in turn to 58, 72, and 64 from 140^26^ before nitrogen doping, indicating that defects were introduced into the carbon framework during NH_3_ treatment, and these may act as active sites for catalysis.

To identify N‐doping of the carbon covering layer and the SWCNTs after NH_3_ treatment, XPS characterization was performed. Figure S2 of the Supporting Information shows survey spectra of the three NCNi/SWCNT films, indicating the presence of nickel, carbon, oxygen, and nitrogen, and their contents are shown in Table S1 of the Supporting Information. High‐resolution XPS spectrum of NCNi/SWCNT‐700 (Figure 3b) for Ni 2p confirms the existence of metallic Ni species which is consistent with the TEM and XRD results. Figure 3c shows the high‐resolution XPS spectrum of NCNi/SWCNT‐700 for C 1s, which can be fitted by three peaks at 284.7, 285.7, and 288.6 eV, corresponding to C—C, C—N, and C=O bonds, respectively. Furthermore, the N 1s spectrum of NCNi/SWCNT‐700 (Figure 3d) fitted by three peaks at 398.5, 400.5, and 402.1 eV confirms the existence of pyridinic, pyrrolic, and graphitic nitrogen in the sample.^32^, ^33^, ^34^ Figure 3e shows an SEM image of NCNi/SWCNT‐700 and the corresponding EDS elemental maps of C, N, and Ni (Figure 3f–h), indicating the uniform doping of N into the structure. XPS spectra of the films before N doping (CNi/SWCNT) are shown in Figure S3 of the Supporting Information, where we can see the presence of C, Ni, and O in the sample while no N signal was detected. The NCNi/SWCNT self‐standing film with a 3D SWCNT conductive network, small Ni nanoparticles encapsulated by single layer of N‐doped carbon, and intimate contact between Ni particles and SWCNT network is expected to show excellent OER and HER catalytic ability.

**Figure 4:**
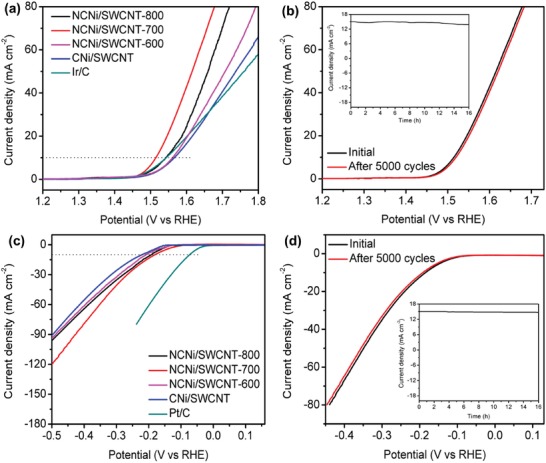
a) OER polarization curves of the NCNi/SWCNT and CNi/SWCNT catalysts with Ir/C for comparison. b) OER durability measurement of NCNi/SWCNT‐700: polarization curves recorded initially and after 5000 cycles. c) HER polarization curves of the NCNi/SWCNT and CNi/SWCNT catalysts with Pt/C for comparison. d) HER durability measurement of NCNi/SWCNT‐700: polarization curves recorded initially and after 5000 cycles. The insets in (b) and (d) are the chronoamperometric curves of NCNi/SWCNT‐700 at a constant potential for OER and HER, respectively.

Electrochemical measurements were carried out in a 1 m KOH solution using a three‐electrode system under ambient conditions. To investigate the catalytic performance of our samples for OER, linear sweep voltammetry (LSV) was used at a scan rate of 5 mV s^−1^. The polarization curves are shown in **Figure** **4**a. Usually, the OER activity of a material is evaluated by the potential required to oxidize water at a current density of 10 mA cm^−2^, which is a metric relevant to solar fuel synthesis.^35^ It can be seen that our NCNi/SWCNT‐700 sample shows a very low onset potential of 1.46 V and an overpotential of 270 mV to reach a current density of 10 mA cm^−2^, which is much better than other catalysts including a commercial Ir/C catalyst. The corresponding Tafel plots are shown in Figure S4a of the Supporting Information. NCNi/SWCNT‐700 has a smaller Tafel slope (56 mV dec^−1^) than the benchmark Ir/C catalyst and other NCNi/SWCNT samples, suggesting its high reaction kinetics for OER.^36^ Furthermore, its stability was tested at a scan rate of 100 mV s^−1^ for 5000 cycles during which there was a negligible change in its polarization curve (Figure 4b). A continuous long‐time (16 h)test at a constant potential of 1.52 V was also performed using chronoamperometry and the results (inset of Figure 4b) show that the catalyst had excellent stability and catalytic activity in alkaline media during this time. We further carried out TEM, XRD, and XPS characterizations of NCNi/SWCNT‐700 after OER stability test to investigate their structure change. TEM image (Figure S5a, Supporting Information) shows that the Ni nanoparticles are still covered by carbon layer after OER stability test and there is no obvious morphology change observed. XRD (Figure S6a, Supporting Information) and XPS (Figure S7a, Supporting Information) characterizations further confirm that Ni particles still maintain their metallic nature after OER stability tests.

We also investigated the HER performance of our samples in comparison with a commercial Pt/C catalyst. Figure 4c shows the LSV polarization curves of various catalysts. Pt/C shows excellent catalytic activity with an overpotential of 70 mV to reach a current density of 10 mA cm^−2^, while NCNi/SWCNT‐700 has an overpotential of 190 mV, which is lower than the other NCNi/SWCNT samples. A smaller Tafel slope value for NCNi/SWCNT‐700 (140 mV dec^−1^) indicates its favorable catalytic kinetics for HER (Figure S4b, Supporting Information). Moreover, Figure 4d shows little difference in the polarization curves of the NCNi/SWCNT‐700 electrocatalyst before and after 5000 CV cycles, indicating its excellent stability. It also shows excellent catalytic activity for 16 h at a constant potential of 0.216 V (inset of Figure 4d). After HER stability test for 5000 cycles, TEM observation (Figure S5b, Supporting Information) of NCNi/SWCNT‐700 shows that the Ni particles are still well encapsulated by carbon shell. XRD (Figure S6b, Supporting Information) and XPS (Figure S7b, Supporting Information) analysis further confirm that the Ni nanoparticles preserve their metallic structure even after long time, continuous testing. We attribute this excellent stability to the efficient protection effect of the monolayer carbon shell.

The OER and HER catalytic performance of the NCNi/SWCNT samples obtained at different temperatures can be elucidated from their XPS spectra. XPS survey and N 1s spectra of the NCNi/SWCNT samples indicate different contents and types of doped nitrogen (Figure S2 and Table S1, Supporting Information). The NCNi/SWCNT‐600 has a higher N content, while that in NCNi/SWCNT‐800 is very low. The high N content in NCNi/SWCNT‐600 indicates a high density of defects, which results in decreased conductivity.^37^ On the other hand, the low N content in NCNi/SWCNT‐800 indicates that there are limited active sites for OER and HER. NCNi/SWCNT‐700 has a medium N content and pyridinic and graphitic N are dominant and this plays a crucial role in determining its superior catalytic activity (Figure S2c, Supporting Information). We further evaluated the double layer capacitance of each electrode, and the results are shown in Figure S8 of the Supporting Information. The NCNi/SWCNT‐700 film has a relatively larger capacitance of 12.2 mF cm^−2^, suggesting its higher electrochemical active surface area than the other two samples, which is in good agreement with the HER and OER catalytic performance. Furthermore, the Ni 2p XPS spectrum of NCNi/SWCNT‐800 (Figure S9, Supporting Information) shows the presence of Ni^2+^, which indicates that some carbon shells surrounding the Ni nanoparticles might have been destroyed during the high temperature treatment, and the inner Ni particles have become exposed and oxidized. This may be the reason for the poor electrocatalytic performance of NCNi/SWCNT‐800. Hence, optimum N doping of CNi/SWCNT guarantees sufficient active sites, high conductivity, and a high electrochemical active surface area, which result in the excellent HER and OER performance of the NCNi/SWCNT‐700 films. The turnover frequencies (TOFs) of different catalysts for OER were further estimated to reveal their intrinsic catalytic activity (Table S2, Supporting Information). The NCNi/SWCNT‐700 shows the highest TOF value of 0.246 s^−1^, which is ≈2.8–4.5 times higher than those of other catalysts, suggesting its higher catalytic activity after N‐doping at optimized conditions.

The possible mechanism for the electrocatalytic activity of metal particles encapsulated in carbon was proposed in some previous studies.^23^, ^38^, ^39^ It is believed that metal particles transfer electrons to encapsulating carbon layer to tune its electronic structure, which optimizes the free energy required for the adsorption and desorption of reaction intermediates. We also attribute the excellent electrocatalytic activity of the NCNi/SWCNT to the synergistic effects between the tiny Ni nanoparticles and the N‐doped monolayer carbon shells. The tiny Ni nanoparticles with abundant surface atoms can optimize the electronic structure of the encapsulating carbon shell to overcome reaction barriers related to adsorption and desorption steps, resulting in enhanced catalytic activity. N‐doping further tunes the adsorption energy of reactants on the surface of carbon shell and thus increases the overall electrocatalytic activities.^40^ In addition, N‐doping introduces a high concentration of defects in the carbon matrix producing a defect‐rich structure with abundant active sites.^41^ Hence, the uniform distribution of N atoms in the carbon network plays a significant role in improving the overall catalytic performance. It can be seen from Figure 4a,c that the NCNi/SWCNT‐700 sample has low overpotentials of 55 and 26 mV compared to CNi/SWCNT to reach a current density of 10 mA cm^−2^ for OER and HER, respectively.

**Figure 5:**
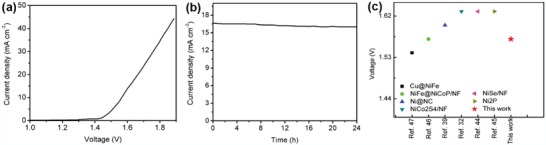
a) LSV curve of NCNi/SWCNT‐700 for overall water splitting at a scan rate of 5 mV s^−1^. b) Chronoamperometric curve at a constant voltage of 1.6 V for NCNi/SWCNT‐700. c) A comparison of applied voltage values to reach a current density of 10 mA cm^−2^ of our NCNi/SWCNT‐700 and previously reported Ni‐based bifunctional electrocatalysts.

Considering the excellent OER and HER activity of the NCNi/SWCNT‐700 catalyst, its bifunctional activity was further investigated for overall water splitting in a 1 m KOH solution using self‐standing rods of NCNi/SWCNT‐700 as both anode and cathode. An optical photograph of the alkaline water electrolyzer setup indicating the evolution of gas bubbles on the surface of the electrodes is shown in Figure S10 of the Supporting Information. The LSV curve (**Figure** **5**a) clearly shows the overall water splitting potential of 1.57 V to achieve a current density of 10 mA cm^−2^, and this is among the best reported values for Ni‐based bifunctional electrocatalysts (Figure 5c).^42^, ^43^, ^44^, ^45^, ^46^, ^47^ For comparison, pairs of Pt/C‐Ir/C and NCNi/SWCNT‐700 electrodes were loaded onto nickel foam and their LSV curves are shown in Figure S11a of the Supporting Information, suggesting the superior catalytic activity of NCNi/SWCNT‐700 compared to the Pt/C‐Ir/C electrolyzer. Figure 5b shows the chronoamperometric curve of NCNi/SWCNT‐700 up to 24 h for water splitting at an applied voltage of 1.6 V indicating its excellent stability. We further compared the stability of a NCNi/SWCNT‐700 pair loaded onto nickel foam with a Pt/C‐Ir/C pair using a constant voltage of 1.6 V (Figure S11b, Supporting Information). It can be seen that 96% of the initial current density was retained for NCNi/SWCNT‐700 after 24 h, while only 72% was retained for the Pt/C‐Ir/C. We attribute this high stability to its unique structure of Ni nanoparticles encapsulated in a monolayer carbon, which prevents the aggregation and atmospheric oxidation of the Ni particles and provides strong interaction with the SWCNT network which acts as conductive channels. We investigated the structure of NCNi/SWCNT‐700 by TEM after a 24 h continuous water splitting test. As shown in Figure S12 of the Supporting Information, the original structure of the carbon covering on the Ni nanoparticles is well preserved, which accounts for the excellent stability of the NCNi/SWCNT‐700 catalyst.^39^, ^48^, ^49^, ^50^

The excellent electrocatalytic performance of NCNi/SWCNT‐700 can be attributed to the synergetic effect of small Ni particles, monolayer N‐doped carbon cover, and high‐quality N‐doped SWCNTs (Figure 2b). The small Ni particles with a mean diameter of 2.7 nm provide abundant active sites. The monolayer carbon coating on the Ni nanoparticles not only prevents them from aggregation but also provides desired access to reactants, which results in excellent long‐term catalytic stability. At the same time, it provides strong interaction with the walls of the SWCNTs leading to easy electron transfer. The N‐doping tailors the electron donors and acceptors of the nanocarbons and thus boosts the catalytic activity of the hybrid catalyst. Such a densely packed interconnected network of highly conductive SWCNTs decorated with Ni particles also provides short charge transport paths. Therefore, this unique hybrid structure with numerous exposed active sites, a stable structure and easy charge transport paths, demonstrates excellent catalytic activity for both HER and OER.

## 3 Conclusion

We have prepared a flexible, self‐standing SWCNT film decorated with small (<4 nm) Ni nanoparticles covered by a thin layer of N‐doped carbon for use as HER and OER catalysts. This hybrid structure takes full advantages of each component: the high content of small Ni nanoparticles provides abundant active sites; the thin N‐doped carbon coating on the Ni nanoparticles prevents their air oxidation and agglomeration and hence improves the overall stability; SWCNTs provide conductive channels for easy electron transport and thus improves the overall electrocatalytic performance. As a result the freestanding NCNi/SWCNT‐700 rods show excellent bifunctional overall water splitting activity and robust stability in an alkaline electrolyzer.

## 4 Experimental Section

*Preparation of CNi/SWCNT*: CNi/SWCNTs were synthesized using an FCCVD technique, which was described in detail in the previous work.^26^

*Preparation of NCNi/SWCNT*: The CNi/SWCNT films were heat treated at different temperatures of 600, 700, and 800 °C for 2 h under a 200 sccm NH_3_ flow to obtain NCNi/SWCNT films and these are respectively denoted as NCNi/SWCNT‐600, NCNi/SWCNT‐700, and NCNi/SWCNT‐800. To avoid possible air oxidation of the Ni nanoparticles, an Ar flow was introduced into the reactor as a protective gas during the whole treatment process.

*Characterizations*: The morphology and structure of the NCNi/SWCNT films were characterized by TEM (Tecnai F20, 200 kV), Raman spectroscopy (Jobin Yvon HR800, with a 632.8 nm He‐Ne laser), XRD (Rigaku diffractometer, Cu Kα radiation), XPS (Escalab 250, Al Kα), and scanning electron microscopy with an EDS detector (Nova NanoSEM 430, 10 kV).

*Electrochemical Tests*: Electrochemical measurements were conducted using a 3‐electrode system (CHI 760 E, CH Instruments) at room temperature in a 1 m KOH solution. The NCNi/SWCNT film was cut to match the area of a glassy carbon electrode (diameter ≈5 mm) to which it was attached by dropping 5 µL of 0.05 wt% nafion solution diluted in ethanol onto the surface. This electrode loaded with ≈0.3 mg cm^−2^ served as the working electrode for both OER and HER tests. A Pt wire/graphite rod and Ag/AgCl were used as counter and reference electrodes, respectively. Reference catalysts of Pt/C (20 wt%) and Ir/C (20 wt%) were prepared by dispersing 2 mg of powder in ethanol containing 0.05 wt% nafion. The amount loaded for both Pt/C and Ir/C catalysts was 0.3 mg cm^−2^. The electrolyte solution was first purged with N_2_/O_2_ before any measurements. HER and OER activities were measured by LSV at a scan rate of 5 mV s^−1^ and a rotation rate of 1600 rpm. All the potentials were converted to a reversible hydrogen electrode using the following equation

(1) $$E\left(\mathrm{RHE}\right)=E\left(\mathrm{Ag}/\mathrm{AgCl}\right)+0.059\mathrm{PH}+0.197$$

For overall water splitting test, the NCNi/SWCNT film was rolled into a freestanding rod‐like structure and two such rods acting as anode and cathode were dipped into a 1 m KOH solution. Finally, the LSV curve for overall water splitting was obtained at a scan rate of 5 mV s^−1^.

## Conflict of Interest

The authors declare no conflict of interest.

## Supporting information

Supplementary
