# Supplementary material for: A Freestanding Single‐Wall Carbon Nanotube Film Decorated with N‐Doped Carbon‐Encapsulated Ni Nanoparticles as a Bifunctional Electrocatalyst for Overall Water Splitting
Source: Adv Sci (Weinh). 2019 Apr 19;6(12):1802177. doi: 10.1002/advs.201802177 (PMC6662082; doi:10.1002/advs.201802177)
Supplement: Supplementary file 1 — Supplementary [file ADVS-6-1802177-s001.pdf]

Copyright WILEY-VCH Verlag GmbH & Co. KGaA, 69469 Weinheim, Germany, 2019.

Supporting Information

for *Adv. Sci.,* DOI: 10.1002/advs.201802177

A Freestanding Single-Wall Carbon Nanotube Film Decorated with N-Doped Carbon-Encapsulated Ni Nanoparticles as a Bifunctional Electrocatalyst for Overall Water Splitting

Abdul Majeed, Peng-Xiang Hou, Feng Zhang, Hassina Tabassum, Xin Li, Guo-Xian Li, Chang Liu,* and Hui-Ming Cheng*

Supporting Information

A Free-Standing Single-Wall Carbon Nanotube Film Decorated with N-Doped Carbon-Encapsulated Ni Nanoparticles as a Bifunctional Electrocatalyst for Overall Water Splitting

Abdul Majeed, Peng-Xiang Hou,* Feng Zhang, Hassina Tabassum, Xin Li, Guo-Xian Li, Chang Liu* and Hui-Ming Cheng

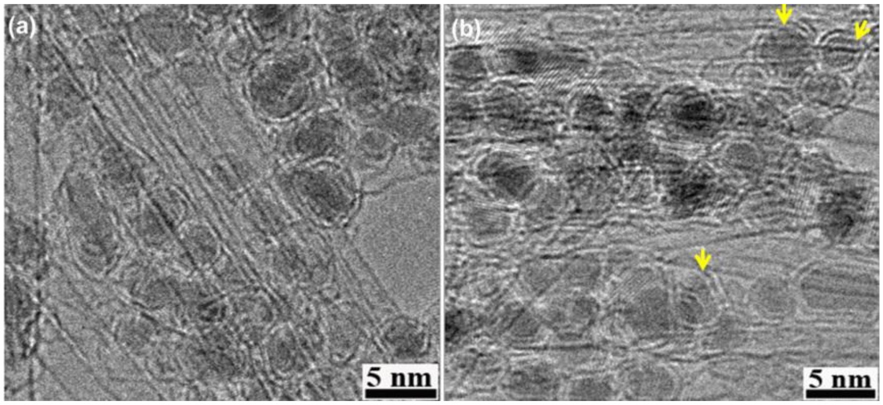

Figure S1. TEM images of a) NCNi/SWCNT-600 and b) NCNi/SWCNT-800 samples.

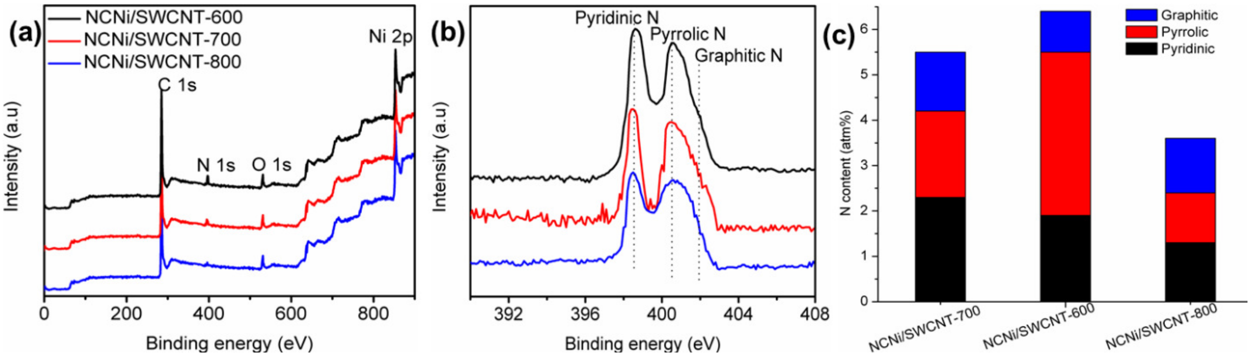

Figure S2. XPS survey spectra of a) NCNi/SWCNT films obtained at different temperatures and b) their corresponding N 1s spectra. c) The contents of different types of N in these samples.

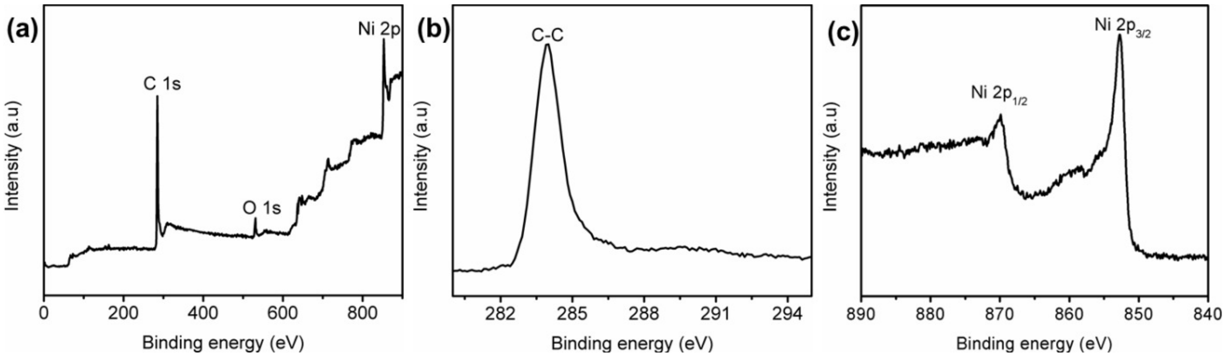

Figure S3. a) XPS survey of CNi/SWCNT sample. b, c) C 1s and Ni 2p spectra of CNi/SWCNT sample, respectively.

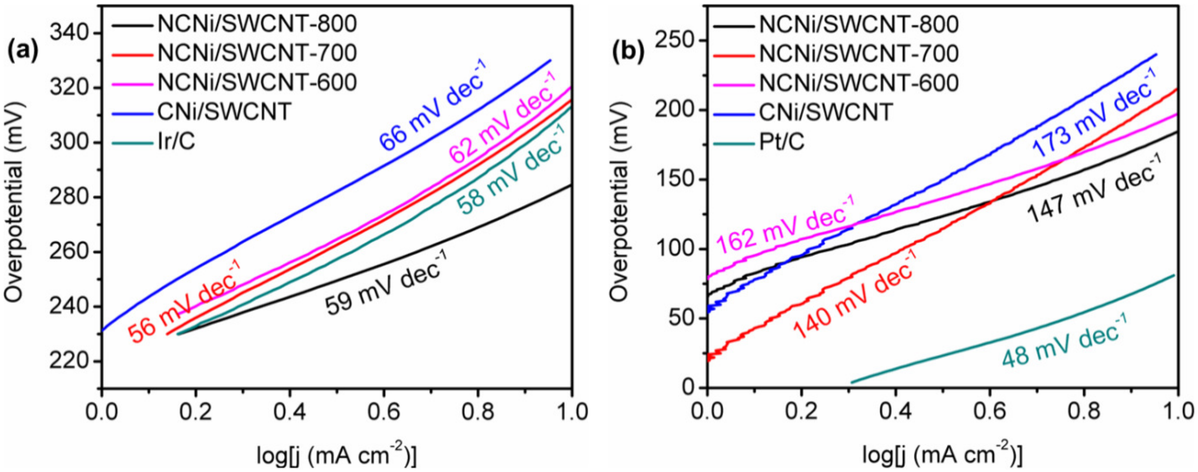

Figure S4. Tafel plots of different catalysts for a) OER and b) HER.

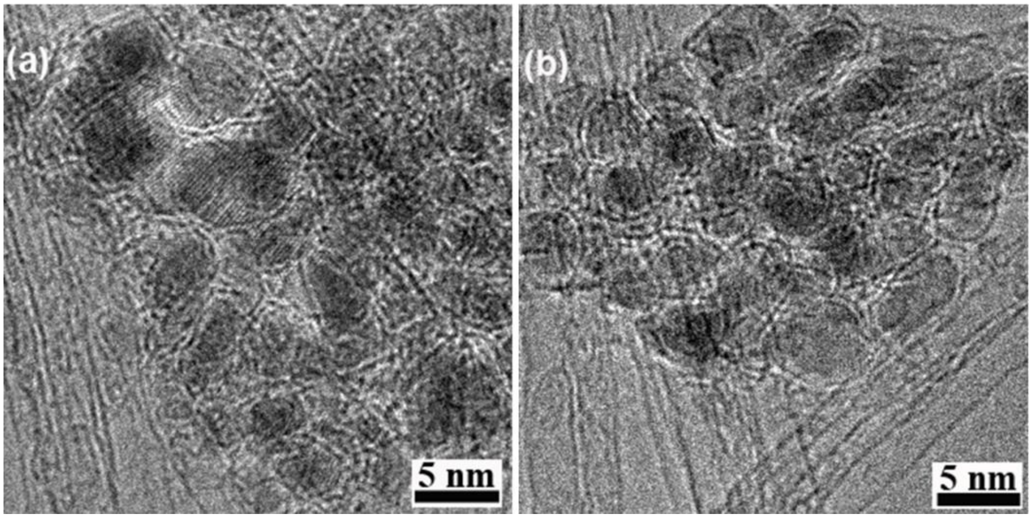

Figure S5. TEM images of NCNi/SWCNT-700 after stability test for a) OER and b) HER.

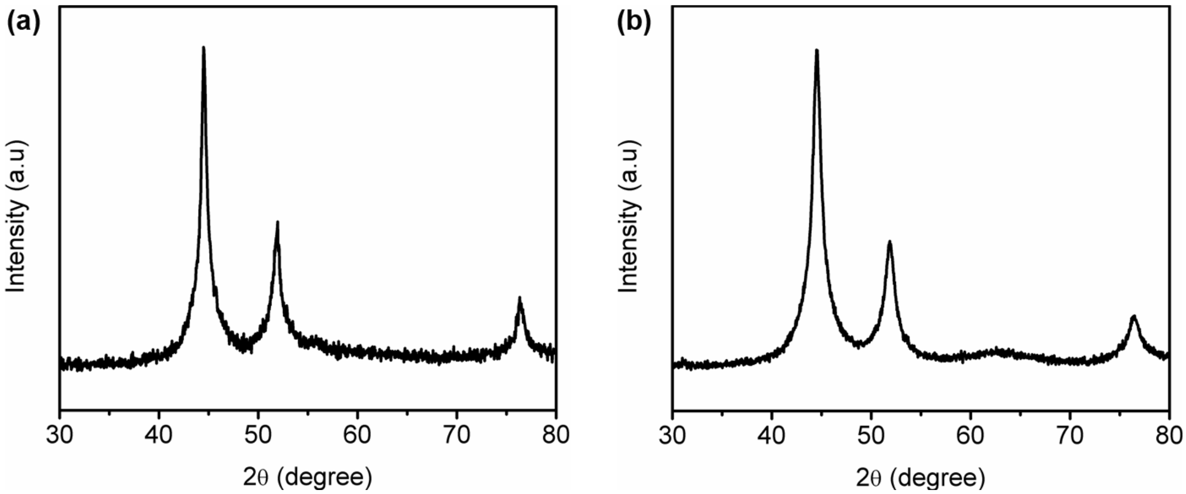

Figure S6. XRD patterns of NCNi/SWCNT-700 after stability test for a) OER and b) HER.

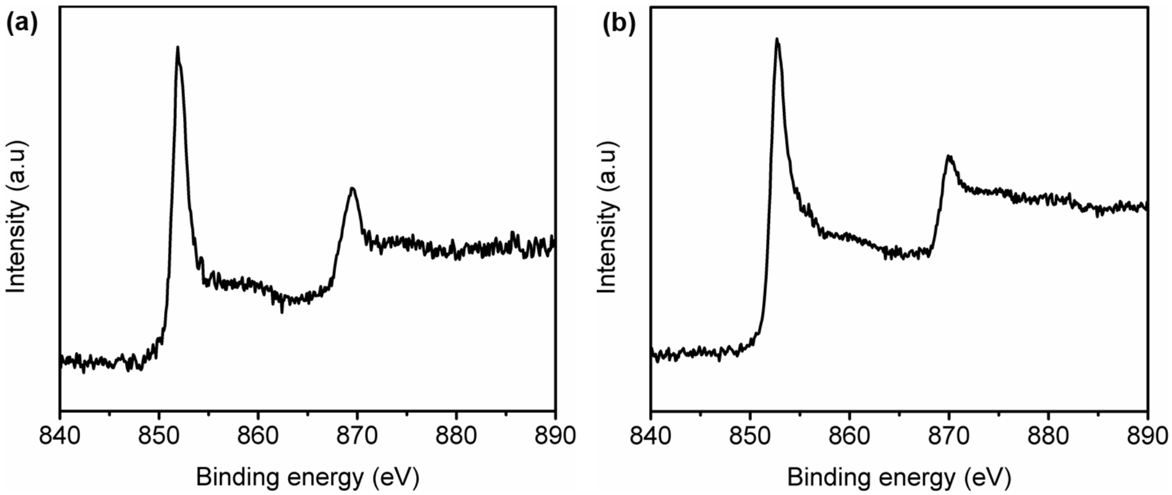

Figure S7. XPS specta of NCNi/SWCNT-700 after stability test for a) OER and b) HER.

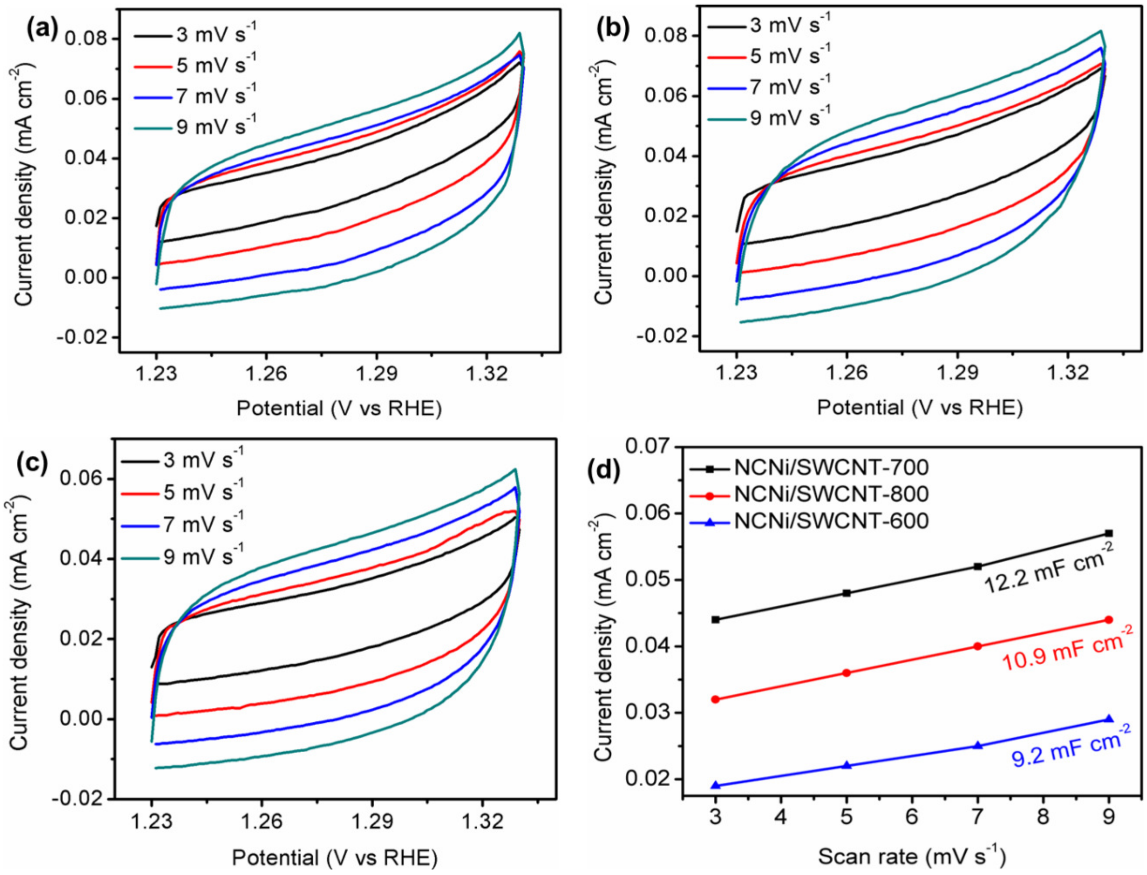

Figure S8. a-c) CV curves of NCNi/SWCNT-700, NCNi/SWCNT-800 and NCNi/SWCNT-600 catalysts at different scan rates, respectively. d) The current density at 1.28 V versus scan rate for the above three samples.

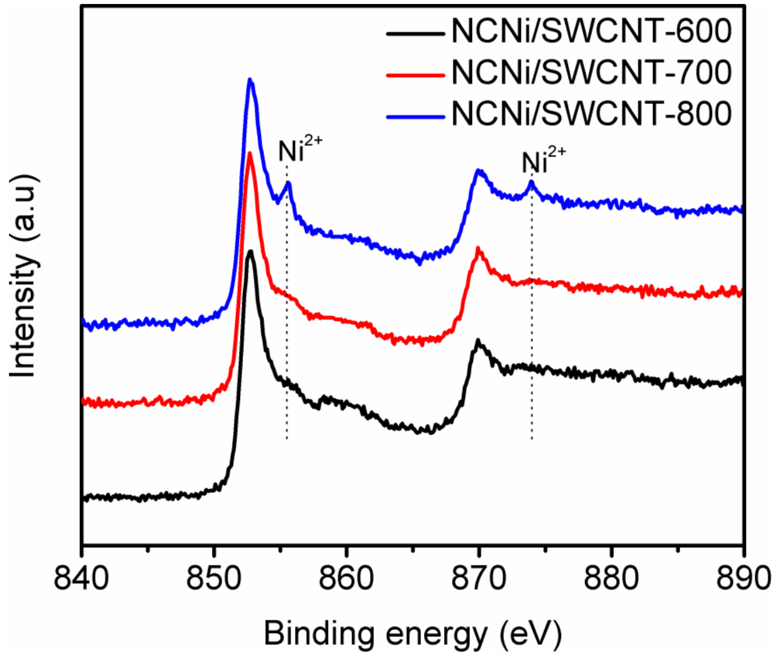

Figure S9. XPS Ni 2p spectra of the NCNi/SWCNT samples.

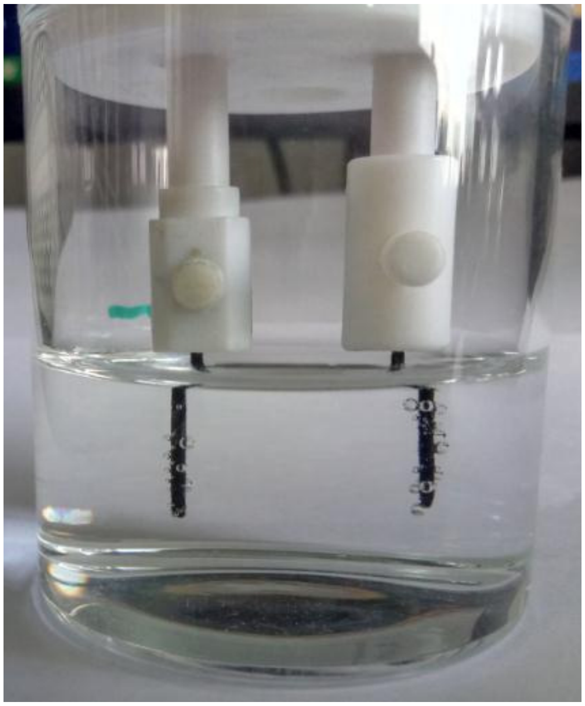

Figure S10. Photograph of alkaline electrolyzer setup using self-standing rods of NCNi/SWCNT-700 as anode and cathode. It can be seen that bubbles appear on the surface of electrodes.

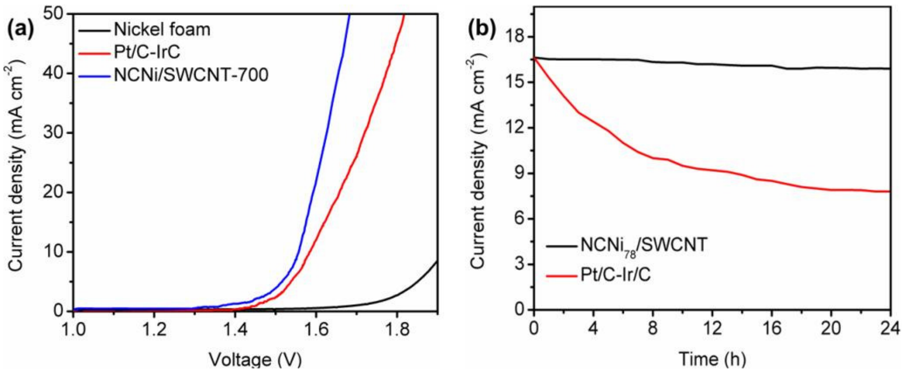

Figure S11. a) LSV curves of Pt/C-Ir/C and NCNi/SWCNT-700 pairs on nickel foam in 1 M KOH solution for overall water splitting at a scan rate of 5 mV s^{-1}. b) Chronoamperometric curve at a constant voltage of 1.6 V for NCNi/SWCNT-700 and Pt/C-Ir/C pairs.

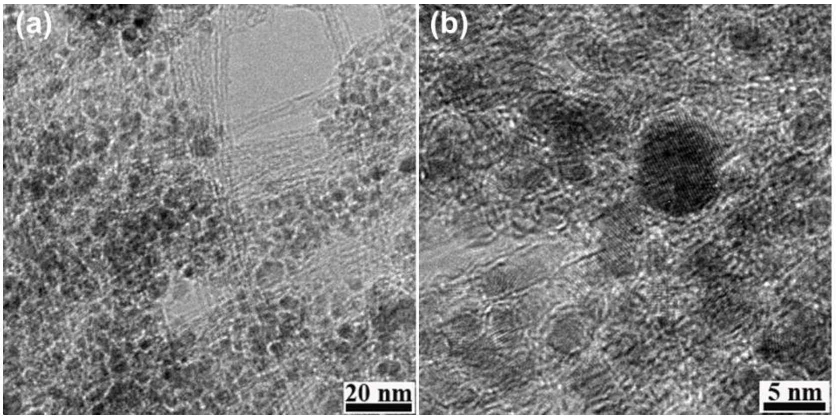

Figure S12. TEM images of NCNi/SWCNT-700 after a 24 h water splitting test.

Table S1. Element contents in the CNi/SWCNT and NCNi/SWCNT samples.

Sample	C (at%)	Ni (at%)	N (at%)	O (at%)
CNi/SWCNT	61.5	33.7	-	4.8
NCNi/SWCNT-600	57.2	31.3	6.4	5.1
NCNi/SWCNT-700	58.7	32.6	3.2	5.5
NCNi/SWCNT-800	59.7	33.2	1.8	5.3

Turnover frequency (TOF) calculations

TOF can be defined as;

$$\text{TOF} = \frac{\text{Number of oxygen turnovr per geometric area}}{\text{Number of active sites per geometric area}}$$

We calculated TOF for OER at an overpotential of 0.35 V in 1M KOH solution assuming that half of Ni particles act as active centers.

TOF can be calculated according to following equation;

$$\text{TOF}\ (s^{-1}) = \frac{jA}{4Fm}$$

where, j is the currnt density at a particular overpotential, A is the surface area, F represents Farady contant and m is the number of moles of active centers (Ni).[1, 2]

Table S2. Estimated turnover frequency values of CNi/SWCNT and NCNi/SWCNT samples.

**Sample**	**TOF (s^{-1})**
CNi/SWCNT	0.054
NCNi/SWCNT-600	0.072
NCNi/SWCNT-700	0.246
NCNi/SWCNT-800	0.088

Supporting references

[1] J. Yin, Q. Fan, Y. Li, F. Cheng, P. Zhou, P. Xi, S. Sun, J. Am. Chem. Soc. **2016**, 138, 14546.

[2] J. Duan, S. Chen, C. Zhao, Nat. Commun. **2017**, 8, 15341.
